# Hemodynamic impacts of flow diverter devices on the ophthalmic artery

**DOI:** 10.1186/s12967-019-1913-4

**Published:** 2019-05-16

**Authors:** Xinzhi Wu, Zhongbin Tian, Jian Liu, Wenqiang Li, Junfan Chen, Yangyang Zhou, Xinjian Yang, Shiqing Mu

**Affiliations:** Department of Interventional Neuroradiology, Beijing Neurosurgical Institute, Beijing Tiantan Hospital, Capital Medical University, Beijing, 100070 China

**Keywords:** Ophthalmic artery, Aneurysm, Flow diverter device, Computational fluid dynamics

## Abstract

**Background:**

Flow diverter devices are increasingly used for endovascular treatment of internal carotid artery aneurysms. Treatment of ophthalmic segment aneurysms with flow diverter devices also includes coverage of the ophthalmic artery but may result in complications. It is unclear, however, whether these devices mechanically block blood flow in the ophthalmic artery. Also unclear is the relationship between deployment of a flow diverter device and post-treatment occlusion. We studied hemodynamic changes in the ophthalmic artery after deployment of a flow diverter device to determine the relationship between those changes and post-stent occlusion of the artery.

**Methods:**

We analyzed hemodynamic modifications in the ophthalmic artery in 21 patients (19 women, 2 men; mean age 53.43 ± 7.32 years) treated by a single pipeline embolization device. Patient-specific geometries were determined from three-dimensional digital subtraction angiography and the stenting process was simulated. Computational fluid dynamics technology was used to analyze the change in ophthalmic artery hemodynamics. We compared pre-treatment and post-treatment flow velocity of the ophthalmic artery.

**Results:**

Among the 21 patients with aneurysms located in the ophthalmic segment, no ophthalmic artery occlusion was found during immediate or follow-up angiography. Post-stent flow velocity in the ophthalmic artery decreased from 0.35 ± 0.19 to 0.33 ± 0.20 m/s, with the difference not being statistically significant (P = 0.106).

**Conclusion:**

Our results showed no significant change in ophthalmic artery blood flow after pipeline embolization device deployment. Hence, post-stent occlusion of the ophthalmic artery could not be explained by reduced blood flow. Delayed thrombosis and neointimal formation maybe the keys to ophthalmic artery occlusion and need further investigation.

**Electronic supplementary material:**

The online version of this article (10.1186/s12967-019-1913-4) contains supplementary material, which is available to authorized users.

## Background

Flow diverter devices have been recognized as important tools to treat intracranial aneurysms [1, 2]. The pipeline embolization device (PED, eV3/Covidien, Irvine, CA) is a flow diverter device approved for use in the treatment of large or giant wide-necked intracranial aneurysms arising from the internal carotid artery between the petrous and hypophyseal segments. Major branches of the internal carotid artery, especially the ophthalmic artery, were often covered by the PED during treatment of ophthalmic segment aneurysms. After PED deployment, aneurysmal flow exhibits reduced fluid dynamic activity [3]. Aneurysms treated with the PED have a high rate of complete occlusion. A recent study reported 86.8% occlusion at 1-year follow-up, which increased to 93.4% and 95.2% at 3- and 5-year follow-ups, respectively [4, 5].

Because the ophthalmic artery can be covered following PED treatment of aneurysms, the patency of the ophthalmic artery is a major concern, as are the clinical results. There are several reports in the literature of ophthalmic artery occlusion and correlated symptoms after PED deployment. Mascitelli et al. [6] described a patient whose ophthalmic artery was covered by PED and occluded immediately. Rouchaud et al. [7] reported that 39.3% patients in their study developed new ophthalmic complications. Limited previous studies have shown that the immediate and delayed occlusion rates of covered ophthalmic arteries range from 5.9 to 21.0% [8–11], but the mechanism of the occlusion was still uncertain, and the post-treatment hemodynamic modification of the ophthalmic artery was unknown.

Aneurysms covered by a PED have a high rate of occlusion, which could be correlated with reduced flow velocity [12]. It is possible that the ophthalmic artery covered by a PED also has reduced flow velocity and other hemodynamic indices related to its post-procedure occlusion. Computational fluid dynamics (CFD) technology is a valid method for evaluating cerebral vascular hemodynamics [13]. In this study, we aimed to identify the hemodynamic changes in the ophthalmic artery induced by PED and the relationship between those changes and the occlusion of the ophthalmic artery.

## Methods

### Study population

From November 2015 to May 2018, 21 subjects with aneurysms were recruited and enrolled in our study. The aneurysms met the following criteria: (1) treated by a single PED with coverage of the ophthalmic artery; (2) unruptured intracranial aneurysm; (3) reconstructed three-dimensional model of the ipsilateral internal carotid artery was available; (4) the landing zone of the PED could be identified by digital subtraction angiography images. Exclusion criteria included a ruptured aneurysm, dissection aneurysm and traumatic aneurysm. The ethics board of our hospital approved the study and all patients agreed to participate and signed informed consent forms. The average age of recruited patients was 53.43 ± 7.32 years. The mean age for women (n = 19) was 53.0 ± 7.16 years and for men (n = 2) 57.5 ± 10.61 years. The mean follow-up time was 6.45 ± 2.83 months. Patient demographics, aneurysm characteristics and treatment strategies displayed in Table 1.

**Table 1:** Patient demographics, aneurysm characteristics and treatment strategies of the patients with coverage of ophthalmic artery after PED deployment

Characteristic	Value
No. of patients	21
Average age (years)	
Male	57.5 ± 10.61
Female	53.0 ± 7.16
Sex	
Male	2
Female	19
Mean aneurysm size (mm)	8.3 ± 4.8
Mean ophthalmic artery diameter (mm)	1.1 ± 0.18
Treatment strategy	
PED	16 (76.2%)
PED + coil	5 (23.8%)
Mean follow-up time (months)	6.4 ± 2.8

### Vessels and stent deployment modeling

**Fig. 1:**
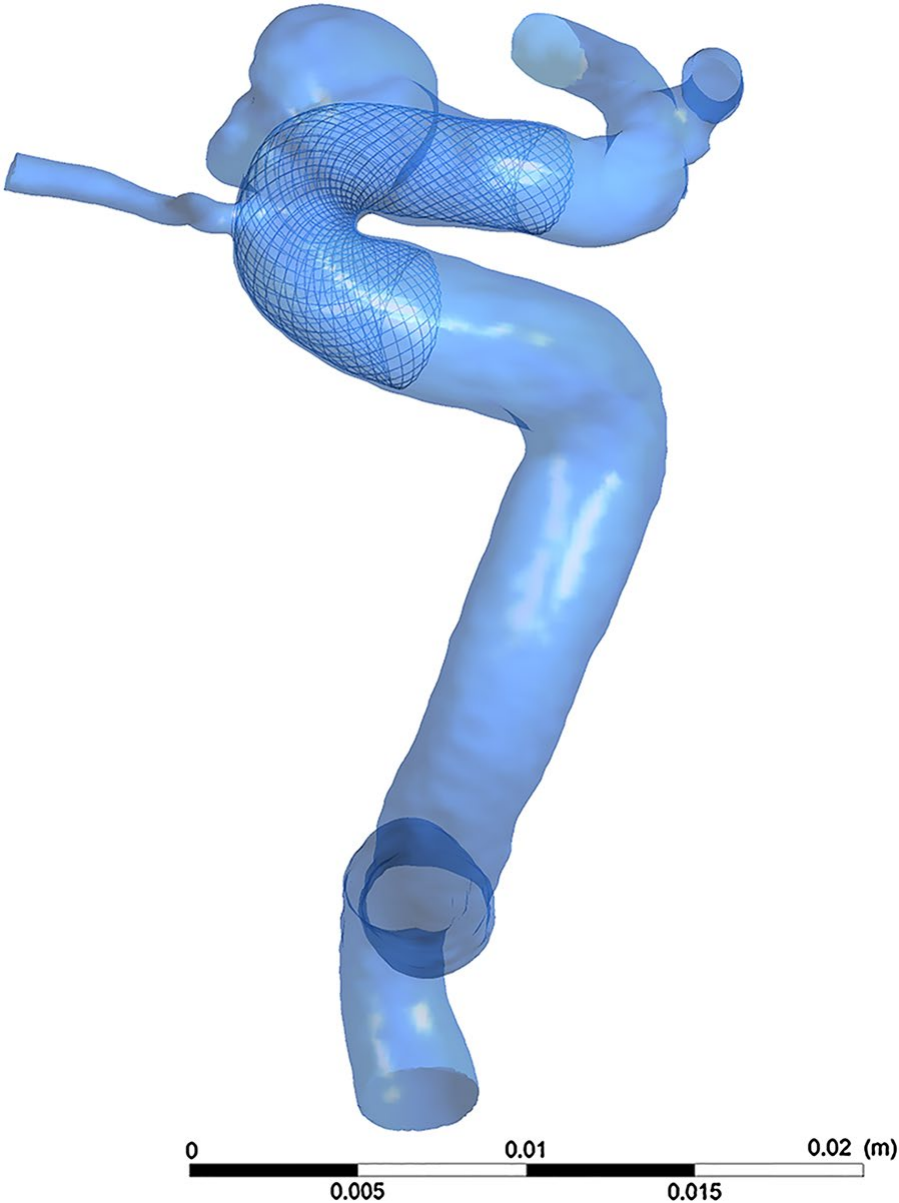
A representative case of the reconstructed vascular model and the virtually implanted PED

The three-dimensional vessel geometries were reconstructed from Digital subtraction angiography images. The images were segmented and smoothed using Geomagic Studio (version 12.0; Geomagic, Research Triangle Park, NC), and the surface geometries were saved as standard tessellation language (STL) format files. The process included three main procedures: (1) pre-processing using vessel-specific initialization to isolate the parent vessel and create simplex mesh that fit the vessel by its centerline; (2) expanding the simplex mesh to make the deployed simplex mesh closely join the wall of the parent vessel; (3) post-processing. The deployed stent was merged with the aneurysm geometry using ICEM CFD version 14.0 (ANSYS, Inc., Canonsburg, PA). Figure 1 displays a representative case of the reconstructed vascular model and the virtually implanted PED.

### CFD modeling

The CFD simulations were as described previously [14, 15], with each aneurysm model imported into the automatic mesh generation software ICEM CFD to create finite-volume element grids for CFD simulations. The maximum element size was set at 0.2 mm. To present the geometry of the pipeline stent sufficiently, the element size on the stent was set at 0.01 mm (approximately one-third the width of the strut of the pipeline stent [16]). A high resolution advection scheme was used in our CFD modeling. Time steps were set at 0.001 s. The untreated cases comprised 1 million tetrahedral elements and the cases treated by PED comprised 5 million tetrahedral elements. To ensure the accuracy of solved flow near walls, we applied three layers of prismatic grid in a hybrid grid near the wall, and applied a tetrahedron grid in the other field. The ANSYS CFX 14.0 was then used to solve the flow-governing Navier–Stokes equations. The blood vessel walls were supposed to be rigid, with no-slip boundary conditions. The blood flow was assumed to have laminar flow (Additional file 1: Table S1) and be a homogeneous, incompressible Newtonian fluid. The dynamic viscosity of the blood flow was set at 0.004 kg/m/s and the density of blood at 1060 kg/m^3^. Using transcranial Doppler imaging, we obtained a representative pulsatile period velocity profile from a normal human, which we used to set the inflow boundary conditions. The pressure distribution along the parent artery, the ophthalmic artery and in the aneurysm were then computed using the drop in pressure calculated during the CFD simulations with respect to P = 10,000 Pa prescribed at the outlet [17, 18]. Flow waveforms were scaled to achieve a mean inlet wall shear stress (WSS) of 1.5 Pa under pulsatile conditions [18]. Traction-free boundary conditions were implemented at the outlet. To avoid initial transient variation, two complete cardiac cycles were computed, and data of the second cycle were gathered.

### Velocity measurement

The flow velocity in the ophthalmic artery was measured at a transection of its ostium at peak systole. Enlargement of the ophthalmic artery’s origin was avoided during the process of obtaining the cut plane. The flow velocity in aneurysms was defined as the average velocity of the entire aneurysm at peak systole.

### Statistical analysis

Statistical analysis was performed with SPSS Statistics for Windows, Version 17.0 (SPSS Inc., Chicago, IL). All quantitative hemodynamic parameters (before and after stent implantation) were summarized as the mean ± SD if normally distributed, and then analyzed with the paired-samples t test. A P value < 0.05 was considered statistically significant.

## Results

The hemodynamic results before and after PED deployment in our patients are shown in Table 2. Eighteen of the 21 treated aneurysms (85.7%) were demonstrated complete occlusion at follow-up. All the 21 (100%) ophthalmic arteries covered by PED were still patent according to angiography performed immediately and at a later follow-up. Two (9.5%) of 21 patients experienced visual symptoms in spite of patency of the ophthalmic artery.

**Table 2:** Summary of the clinical characteristics and hemodynamic results before and after PED deployment in our 21 patients

Case no.	Sex, age (year)	Aneurysm site	Aneurysm size (mm)	Pre-treatment velocity (m s^−1^)		Post-treatment velocity (m s^−1^)		Reduction ratio (%)	
				OphA	Aneurysm	OphA	Aneurysm	OphA	Aneurysm
1	M,50	lc6	5.41	0.12	0.02	0.10	0.00	16.52	80.33
2	F,43	lc6	4.64	0.19	0.05	0.15	0.02	19.86	63.46
3	F,58	lc5	0.96	0.12	0.04	0.08	0.01	32.83	85.72
4	F,62	lc6	3.31	0.66	0.36	0.77	0.30	− 17.63	16.96
5	F,52	rc6	4.31	0.09	0.02	0.08	0.00	11.75	79.89
6	F,42	rc6	5.77	0.34	0.11	0.32	0.04	7.23	67.47
7	F,53	rc6	3.8	0.38	0.22	0.34	0.06	11.09	71.91
8	F,52	lc6	3.15	0.43	0.16	0.38	0.06	11.77	64.12
9	F,48	lc6	5.32	0.35	0.12	0.38	0.04	− 7.51	63.41
10	F,43	lc6	2.24	0.08	0.03	0.07	0.00	5.96	87.85
11	M,65	rc6	5.5	0.50	0.08	0.48	0.03	3.75	64.55
12	F,53	rc6	4.43	0.40	0.20	0.38	0.12	6.13	42.62
13	F,57	lc6	5.55	0.36	0.09	0.31	0.03	12.67	64.28
14	F,62	lc6	8.57	0.49	0.18	0.47	0.10	4.30	45.24
15	F,57	lc5	19.8	0.79	0.03	0.75	0.00	4.98	85.47
16	F,46	rc6	4.31	0.35	0.23	0.34	0.08	2.86	63.83
17	F,58	rc6	4.64	0.44	0.01	0.45	0.00	− 0.34	90.88
18	F,53	rc6	6.09	0.16	0.05	0.16	0.02	− 3.66	65.01
19	F,63	lc5	13.8	0.54	0.07	0.51	0.02	5.36	75.49
20	F,62	rc5	4.95	0.28	0.06	0.27	0.03	4.48	51.91
21	F,43	rc6	21.1	0.16	0.01	0.17	0.01	− 3.76	− 20.83

**Fig. 2:**
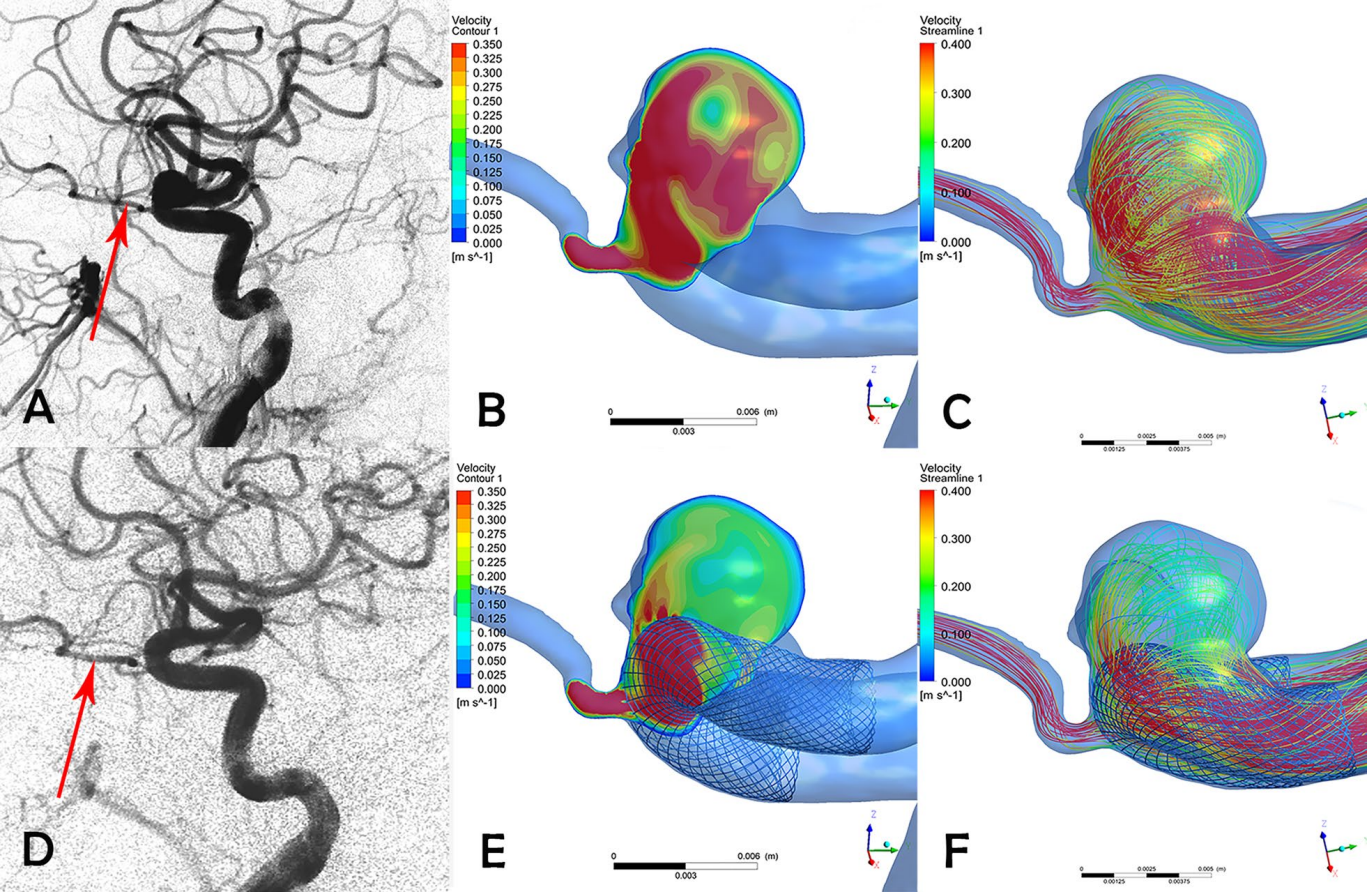
Flow conditions before and after Pipeline embolization device deployment. **a** Pre-stent angiographic image of the aneurysm and ophthalmic artery. **b** Pre-stent velocity magnitude on a cut plane at peak systole. **c** Pre-stent streamline of the aneurysm and ophthalmic artery. **d** Angiographic image at the 6-month follow-up, with complete occlusion of the aneurysm and patency of the ophthalmic artery. **e** Post-stent velocity magnitude on the same cut plane at peak systole. Flow velocity of the ophthalmic artery remains unchanged, whereas flow velocity of aneurysm is obviously reduced. **f** Post-stent streamline of the aneurysm and ophthalmic artery compared with the pre-stent condition. There is no obvious change in the streamline in the ophthalmic artery

**Fig. 3:**
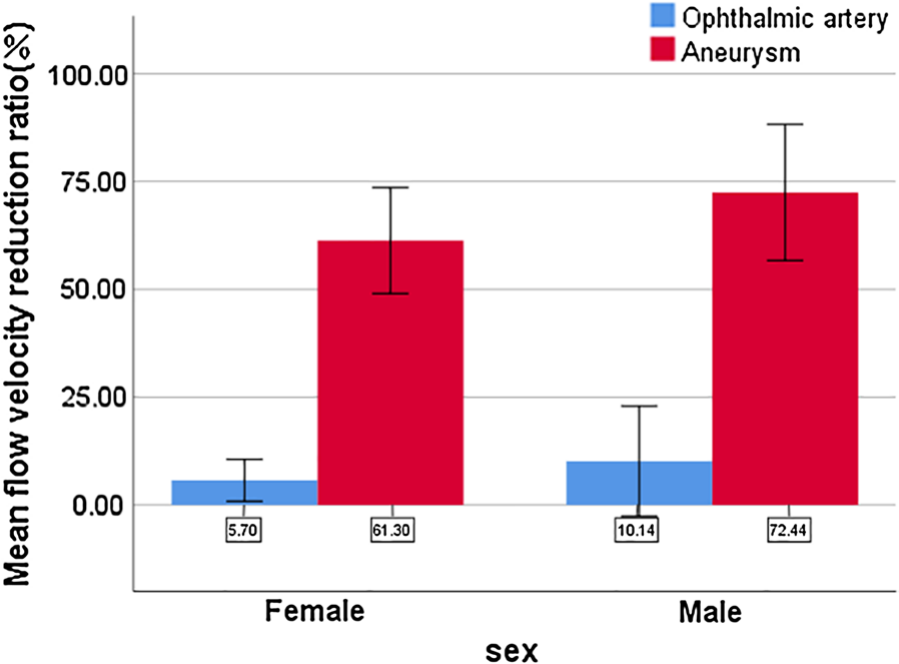
Mean flow velocity reduction ratio in ophthalmic artery versus aneurysm and sex differences

The pre- and post-procedural flow velocity of the ophthalmic artery and intra-aneurysmal flow are shown in Fig. 2. Before deployment of the pipeline stent, the blood flow velocity in the ophthalmic artery was 0.35 ± 0.19 m/s. The post-procedural flow velocity was 0.33 ± 0.20 m/s (P = 0.106). The velocity in the aneurysm significantly decreased, dropping from 0.10 ± 0.09 to 0.05 ± 0.07 m/s (P < 0.001). The flow reduction ratio [(pre-treatment parameter − post-treatment parameter)/pre-treatment parameter] of the ophthalmic artery was 6.13 ± 10.37%. The flow reduction ratio of the aneurysm was 62.36 ± 25.75%. Figure 3 depicts the mean flow velocity reduction ratio in ophthalmic artery versus aneurysm and sex differences.

## Discussion

This study of hemodynamic changes from before deployment of the flow diverter device to afterward showed that flow velocity changes in the ophthalmic artery at peak systole were not significant. The blocking effect of the PED on ophthalmic artery blood flow was limited compared with its blocking effect on the intra-aneurysmal flow velocity. This result is opposite from what we expected—that the metal stent would likely block the blood flow into the ophthalmic artery. We have, for the first time, shown that blood flow in the ophthalmic artery covered by a PED is not reduced as much as it is in the aneurysm, remaining the same as it was before the stent was deployed.

Hu et al. [19] studied blood flow reduction in 31 patients with covered anterior inferior cerebellar arteries using the CFD approach. The group modeled deployment of the flow diverter device into the basilar artery and covered the anterior inferior cerebellar arteries. Flow velocity reduction of each anterior inferior cerebellar artery after deployment of flow diverter device was measured. They reported that blood flow reduction in the anterior inferior cerebellar artery was 3.6175 ± 1.94% and concluded that there was no significant change in the blood flow in the anterior inferior cerebellar arteries due to PED treatment, similar to the results of our study.

Ouard et al. [12] studied the relationship between post-stent blood flow reduction and complete occlusion of aneurysms by means of CFD analysis. Hemodynamic characteristics of intra-aneurysmal flow with and without a virtual stent were compared. They concluded that to achieve complete occlusion of the aneurysm, blood flow velocity in sidewall aneurysms must be reduced at least one-third from pre-stent velocities. There may also be a minimum blood flow reduction threshold for post-stent ophthalmic arteries. In our study, the blood flow reduction after deployment of a single pipeline stent apparently did not reach this minimum threshold. This hypothesis should be confirmed in a subsequent study.

In our prior research, Wang et al. [20] studied 10 miniature pigs to investigate the flow obstruction effect of the PED, Low-profile Visualized Intraluminal Support (LVIS) and Solitaire stents on blood flow in collateral arteries. Stents were inserted into an internal carotid artery segment of miniature pigs that covered at least one collateral branch. Angiography was performed immediately after stent deployment and obstruction of blood flow to brain were evaluated by magnetic resonance imaging. Stents were examined to find evidence of neointima formation. No obstruction was found in the collateral branches and neointima formation were observed in the stents. Dai et al. [21] conducted an in vivo study to investigate patency of branches after coverage with multiple telescoping PEDs. Rabbits were divided into 3 groups (single, double and triple PED). Follow-ups were conducted at 6 and 12 months. All the covered branches were patent at follow-up. However, partial neointimal coverage of ostia of the branches was seen. As the neointima was not continuous, investigators observed prompt flow into the branches from the aorta during a saline flush. We speculate that the ostia of the branches would be completely covered by neointima with a longer duration follow-up. Kulcsar et al. [22] postulated that neointimal hyperplasia was a possible explanation for delayed infarctions in their 3 patients. The theories above can explain why some ophthalmic arteries became occluded without flow velocity changes soon after PED deployment.

The reports in the literature for post-treatment occlusion rate of ophthalmic arteries ranging from 5.9 to 21.0% [8–11] were perplexing because we found no ophthalmic artery occlusion. We think the difference is because of the enrollment process. In order to eliminate the influence of a coil on the accuracy of the results, we enrolled stent-only cases and specifically those with an ophthalmic artery originating from a parent artery. Perhaps there is less post-treatment flow reduction in the ophthalmic artery without coils. Further study of this issue is warranted.

The present study confirms that there is no significant change in ophthalmic artery blood flow. However, this study has several limitations. First, only 21 patients with ophthalmic artery covered were recruited in our study. This limited number of subjects may affect the accuracy of our results. Second, blood vessels walls were assumed to be rigid, which might not represent the true physiology of the human body. Third, no patients with occluded ophthalmic arteries were enrolled in our study, and more persuasive results might have been obtained with such cases. Finally, blood is modeled as a Newtonian fluid with laminar flow, which may affect the hemodynamic results.

## Conclusion

This is the first study analyzing blood flow changes in the ophthalmic artery using CFD technology. We found that PEDs exerted little to no influence on blood flow in the ophthalmic artery; at the very least, the influence was not as great as believed. It is likely that hemodynamic changes such as blood flow reduction are not responsible for the occlusion and other complications of the ophthalmic artery after pipeline stent deployment. Formation of neointima maybe responsible for this occlusion and requires more in-depth investigation (Additional file 1: Table S1).

## Additional file

**Additional file 1: Table S1.** Reynolds value and proves this assumption.

## Data Availability

All clinical data related to this study are stored by an information system at the Department of Interventional Neuroradiology, Beijing Neurosurgical Institute and Beijing Tian Tan Hospital, Capital Medical University.

## Abbreviations

CFD: computational fluid dynamics
PED: pipeline embolization device
