# Supplementary material for: Hemodynamic impacts of flow diverter devices on the ophthalmic artery
Source: J Transl Med. 2019 May 16;17:160. doi: 10.1186/s12967-019-1913-4 (PMC6524319; doi:10.1186/s12967-019-1913-4)
Supplement: Supplementary file 1 — Additional file 1: Table S1. Reynolds value and proves this assumption. [file 12967_2019_1913_MOESM1_ESM.docx]

Table S1. Reynolds values observed at ostium of ophthalmic artery and within the aneurysm

| Case No. | Pre-treatment | | Post-treatment | |
| --- | --- | --- | --- | --- |
|  | Near the ostia of ophthalmic artery | Within the aneurysm | Near the ostia of ophthalmic artery | Within the aneurysm |
| 1 | 41 | 200 | 34 | 94 |
| 2 | 75 | 130 | 60 | 72 |
| 3 | 25 | 37 | 17 | 12 |
| 4 | 195 | 347 | 230 | 326 |
| 5 | 22 | 72 | 20 | 28 |
| 6 | 127 | 593 | 118 | 375 |
| 7 | 126 | 427 | 112 | 239 |
| 8 | 129 | 216 | 114 | 170 |
| 9 | 154 | 654 | 165 | 314 |
| 10 | 29 | 118 | 27 | 52 |
| 11 | 202 | 447 | 195 | 295 |
| 12 | 131 | 370 | 123 | 326 |
| 13 | 92 | 283 | 81 | 178 |
| 14 | 159 | 929 | 152 | 627 |
| 15 | 267 | 754 | 253 | 304 |
| 16 | 108 | 455 | 105 | 294 |
| 17 | 162 | 81 | 163 | 43 |
| 18 | 47 | 489 | 49 | 139 |
| 19 | 197 | 886 | 186 | 269 |
| 20 | 113 | 241 | 108 | 175 |
| 21 | 57 | 543 | 59 | 317 |
